# Supplementary material for: Temperature and magnetic field dependence of a Kondo system in the weak coupling regime
Source: Nat Commun. 2013 Jul 1;4:2110. doi: 10.1038/ncomms3110 (PMC3730050; doi:10.1038/ncomms3110)
Supplement: Supplementary Information — Supplementary Figures S1-S4, Supplementary Notes 1-3 and Supplementary References [file ncomms3110-s1.pdf]

Supplementary Information for 'Temperature and Magnetic Field Dependence of a Kondo System in the Weak Coupling Regime'

Yong-hui Zhang,[1,2,*] Steffen Kahle,[1,*] Tobias Herden,[1] Christophe Stroh,[3] Marcel Mayor,[3,4] Uta Schlickum,[1] Markus Ternes,[1,†] Peter Wahl,[1,5,‡] and Klaus Kern[1,6]

[1]*Max-Planck Institute for Solid State Research, Heisenbergstraße 1, 70569 Stuttgart, Germany*

[2]*Department of Physics, Tsinghua University, Beijing 100084, China*

[3]*Institute of Nanotechnology, Karlsruhe Institute of Technology, P.O. Box 3640, 76021 Karlsruhe, Germany*

[4]*Department of Chemistry, University of Basel, 4056 Basel, Switzerland*

[5]*SUPA, School of Physics and Astronomy, University of St. Andrews, St.Andrews, KY16 9SS, UK*

[6]*Institut de Physique de la Matière Condensée, Ecole Polytechnique Fédérale de Lausanne (EPFL), 1015 Lausanne, Switzerland*

(Dated: June 5, 2013)

[*]These authors contributed equally

[†]Electronic address: `m.ternes@fkf.mpg.de`

[‡]Electronic address: `wahl@fkf.mpg.de`

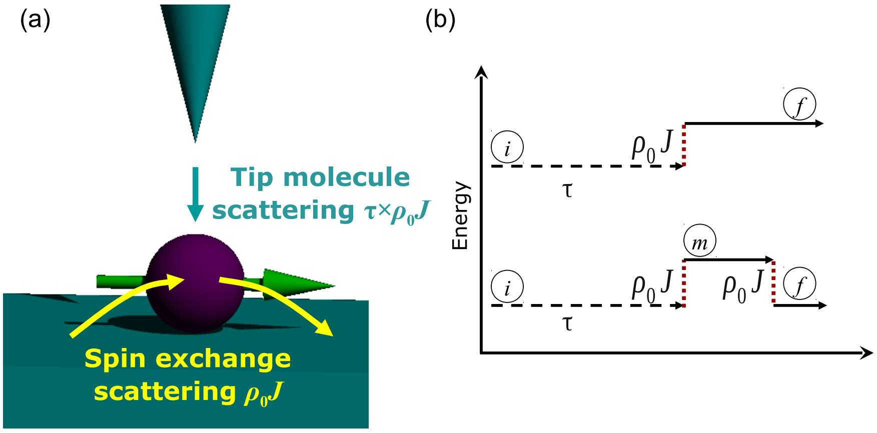

Supplementary Fig. S1: **The tunneling process between tip and sample.** (a): In our model we account for inelastic electron tunneling of strength $\tau \cdot \rho_0 J$ and spin exchange scattering between the localized spin of the molecule and the electrons of the substrate of strength $\rho_0 J$ with ρ_0 as the density of states in the sample at Fermi energy, J as the coupling strength, and τ as the hopping matrix element for an electron from tip to the localized spin system. (b) Scheme of the processes contributing to the shape of the tunneling spectra.

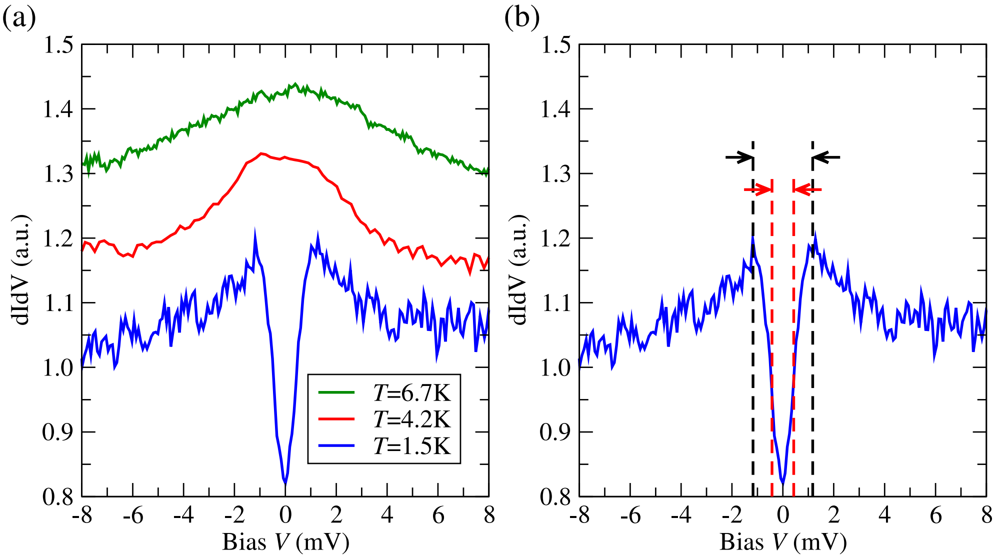

Supplementary Fig. S2: **Temperature dependence of spectra in magnetic field and determination of the splitting** (a) Example spectra measured at a magnetic field of $B = 4$ T and at different temperatures. The split peak is clearly visible at $T = 1.5$ K. At slightly elevated $T = 4.2$ K the splitting is only barely visible and at $T = 6.7$ K completely smeared out. As soon as the thermal energy increases to roughly $E_Z \approx k_B T$ the split peak can hardly be detected as seen at $T = 4.2$ K ($k_B T = 0.36$ mV). At even higher temperatures, where $E_Z < k_B T$ only one peak is seen in the spectra as shown for $T = 6.7$ K ($k_B T = 0.58$ mV). (b) Determination of the peak splitting at $B = 4$ T and $T = 1.5$ K. The determination of the peak splitting is not straightforward and can lead to an erroneous measurement of the effective g-value. Using the peak positions (dashed black lines) results in a splitting of 2.35 mV and a much too high effective $g = 5.1$. Using the steepest slope (dashed red lines) gives a better estimate of 0.85 mV and an effective $g = 1.8$. Both values differ significantly from the value obtained by fitting the data to the perturbation model (eq. S3) which results in $g = 2.1 \pm 0.2$.

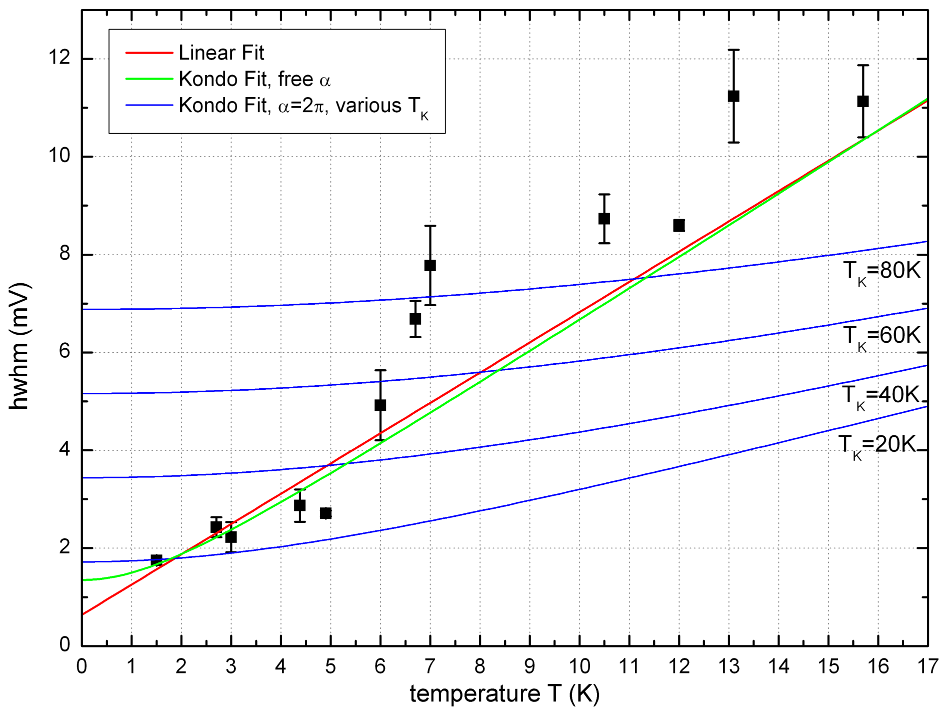

Supplementary Fig. S3: **Temperature dependence of the resonance width.** Black squares denote the width extracted by fitting a Frota-function corrected for the broadening due to the finite tip temperature. The family of blue curves show the expected behaviour in Fermi Liquid theory (eq. S6) with $\alpha = 2\pi$ and different T_{K}.

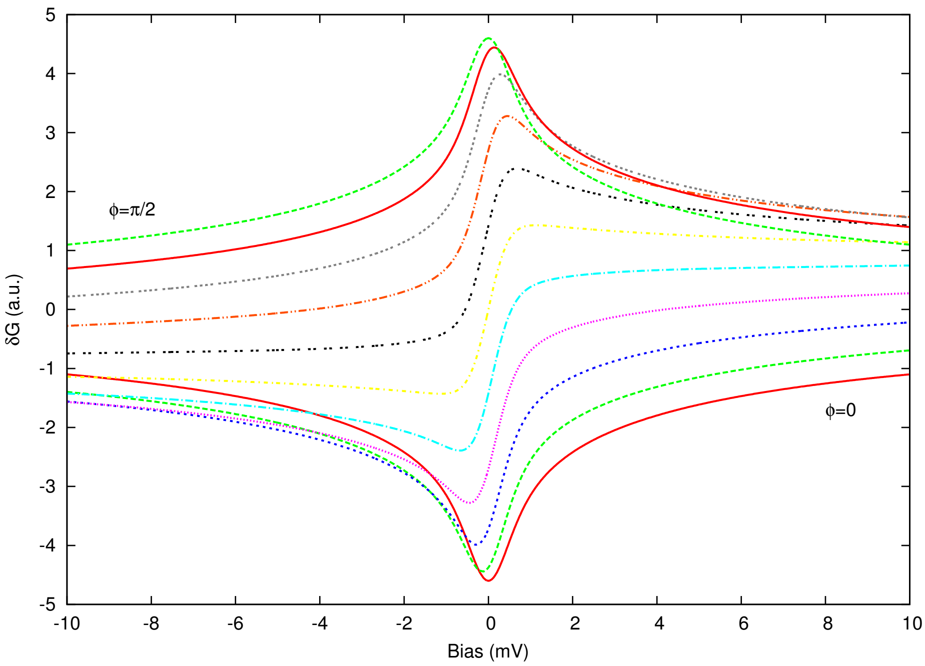

Supplementary Fig. S4: **Differential Conductance for the tip weakly coupled to the impurity state.** Change of differential conductance $\delta G(V)$ at $T = 1.8$K obtained from eq. S9 and accounting for the additional thermal broadening due to the tunneling process as a function of coupling parameter $q = \tan\phi$ for $\phi = 0 \ldots \pi/2$ (normalized by $q^2 + 1$). The line shape changes from a dip to a peak. The line shape becomes a step function for $q = 1$ ($\phi = \pi/4$). The case $q \approx 0$ is encountered for adatom systems on metal surfaces [13, 14], where the impurity spin is localized in a d-orbital.

Supplementary Note 1. THE PERTURBATION MODEL

To describe the excitation processes during tunneling we use a model based on the perturbative approach established by Appelbaum, Anderson, and Kondo [1, 17, 18, 19] in which spin-flip scattering processes up to 3rd order in the spin-spin exchange coupling J are accounted for (see Supplementary Figure S1). In this model the transition probability $W_{i\to f}$ for an electron to tunnel between tip and sample is

$$W_{i\to f} = \frac{2\pi}{\hbar}\left(\tau^2(\rho_0 J)^2|M_{i\to f}|^2 + \tau^2(\rho_0 J)^3\sum_m\left(\frac{M_{i\to m}M_{m\to f}M_{f\to i}}{E_i - E_m} + \text{c. c.}\right)\right)\delta(E_i - E_f), \tag{S1}$$

with $M_{i\to j}$ as the matrix elements given by the interaction of the scattering electron with the localized spin of the molecule

$$M_{i\to f} = \langle\varphi_f, \psi_f|\mathbf{S}_\varphi\cdot\mathbf{S}_\psi|\varphi_i, \psi_i\rangle. \tag{S2}$$

Here, $|\varphi, \psi\rangle$ is the combined state vector of the localized spin and the electron and $\mathbf{S}_{\varphi,\psi} = \{\hat\sigma_x, \hat\sigma_y, \hat\sigma_z\}$ are the standard Pauli matrices for a spin-1/2 system. The eigenstates and energies of the localized spin of the molecule are given by the Zeeman Hamiltonian $\hat H = 2g\mu_\text{B}\mathbf{S}_\varphi\cdot\mathbf{B}$ with g as the gyromagnetic factor, μ_B the Bohr magneton, and $\mathbf{B}$ the external magnetic field.

In this model the tunneling conductance $\sigma(eV) = \mathrm{d}I/\mathrm{d}V(V)$ due to the electron-spin interaction has the following contributions:

$$\begin{aligned}
\sigma_1(eV) =& c\left(\frac{1}{3} + \frac{2}{3}\sum_{\substack{i,f\\ i\neq f}}\varrho_i(T)\left[\Theta\left(\frac{\Delta_{if}+eV}{k_\text{B}T}\right) + \Theta\left(\frac{\Delta_{if}-eV}{k_\text{B}T}\right)\right]\right)\\
\sigma_2(eV) =& c\frac{4\rho_0 J}{3}\sum_{\substack{i,f\\ i\neq f}}\varrho_i(T)\left\{\left[\Theta\left(\frac{\Delta_{if}+eV}{k_\text{B}T}\right) + \Theta\left(\frac{\Delta_{if}-eV}{k_\text{B}T}\right)\right]g\,(eV)\right.\\
&\left. + \left[\Theta\left(\frac{\Delta_{if}+eV}{k_\text{B}T}\right) + \frac{1}{2}\right]g\,(\Delta_{if}+eV) + \left[\Theta\left(\frac{\Delta_{if}-eV}{k_\text{B}T}\right) + \frac{1}{2}\right]g\,(\Delta_{if}-eV)\right\}
\end{aligned} \tag{S3}$$

Here, $c \propto \tau^2(\rho_0 J)^2$ is a constant, $\varrho_i(T) = Z^{-1}\exp[E_i/(k_\text{B}T)]$, with $Z = \sum_i\exp[E_i/(k_\text{B}T)]$, is the thermal occupation of the localized spin in the state i, $\Theta(\epsilon) = [1 + (\epsilon - 1)\exp(\epsilon)][1 - \exp(\epsilon)]^{-2}$ is the thermally broadened step function [34], $\Delta_{if} = E_f - E_i$ is the energy difference

between the spin states of the molecule, and $g(\epsilon)$ the function originating from the divergence of the second term in equation S1. In our model we numerically evaluate $g(\epsilon)$ as

$$g(\epsilon) = \int_{-\omega_0}^{\omega_0} \frac{f(\epsilon', T)}{\epsilon - \epsilon'} \mathrm{d}\epsilon' * f'(\epsilon, T), \tag{S4}$$

with $f(\epsilon, T) = [1 + \exp(\epsilon/k_\mathrm{B}T)]^{-1}$ as the Fermi-Dirac distribution and $f'(\epsilon, T) = \partial f/\partial\epsilon = (k_\mathrm{B}T)^{-1}\mathrm{sech}^2[\epsilon/(2k_\mathrm{B}T)]$ as its derivation [35]. We found that the value of the cut-off energy ω_0 is not critical for the data evaluation. We used $\omega_0 = 20$ mV throughout our data evaluation [24]. Variations of ω_0 between $10 - 200$ mV only slightly changes the coupling $J\rho_0$.

At zero field the conductance simplifies to $\sigma(eV) = \sigma_0 - \alpha \times g(eV/(k_\mathrm{B}T))$ leaving the temperature T as the only relevant fit parameter. We note that the ratio between the intensity of the many-particle state α and the background conductance σ_0 varies strongly with the tip position on the molecule but reaches up to 30%.

At $B \neq 0$ the step-like structure is governed by ordinary inelastic spin-flip scattering of σ_1 in equation S3 [12, 36]. The additional logarithmic peaks in the conductance result from the different possible higher order scattering paths described by σ_2. We determine $\rho_0 J$ by analyzing the intensity of these peaks.

Here, we note that the perturbative approach only holds as long as higher order contributions can be neglected, i. e. $\omega_0 \exp[-(J\rho_0)^{-1}] \ll k_\mathrm{B}T$, that is, as long as the temperature is high compared to the Kondo temperature T_K of our system.

Supplementary Note 2. ALTERNATIVE FITTING MODEL USING THE FROTA FUNCTION

As alternative fitting model for the temperature dependent data we use the Frota function [22]:

$$\tilde{g}(eV) = \Re\sqrt{\frac{i\Gamma_\mathrm{F}}{i\Gamma_\mathrm{F} + eV}}, \tag{S5}$$

which has been successfully used to describe experimental AFM Kondo data [28, 37]. Applying the model on our data yields overall good fits at elevated temperatures, but at low temperature the fit quality deviates significantly (Figure 3a main text).

Additionally, the temperature dependence of the HWHM (half-width at half-maximum) $\Gamma = 2.54 \times \Gamma_\mathrm{F}$ extracted from the Frota-fits does not match the predictions of a strong cou-

pling AFM-Kondo system, for which the Frota function is designed. In the strong coupling limit the peak width Γ at $T = 0$ K defines the Kondo temperature $T_\mathrm{K} = \Gamma/k_\mathrm{B}$, while at finite temperatures the peak broadens in the Fermi-liquid approximation to

$$\Gamma = \frac{1}{2}\sqrt{(\alpha k_\mathrm{B} T)^2 + (2k_\mathrm{B}T_\mathrm{K})^2} \tag{S6}$$

with $\alpha = 2\pi$ [31].

Since equation S6 describes only the broadening of the Kondo feature itself and does not account for the finite temperature of the tip involved in the measurement, we correct the width by a simple quadratic approximation: $\tilde{\Gamma} = \sqrt{\Gamma^2 - \Gamma_\mathrm{tip}^2}$ with $\Gamma_\mathrm{tip} = \frac{1}{2}3.5k_\mathrm{B}T$. As can be seen in figure 3d of the main text and Supplementary Figure S3 the increase of the apparent width $\tilde{\Gamma}$ over temperature is much stronger than expected from the AFM strong coupling model (Eq. S6). Estimating the Kondo temperature by extrapolating the width to zero temperature where we let α be a free fit parameter results in an unphysically high $\alpha = 15.2 \pm 0.9$ and $T_\mathrm{K} = 15.7 \pm 2.5$ K.

Since a $S = 1/2$ AFM Kondo resonance is not expected to split under magnetic fields up to $g\mu_\mathrm{B}B \approx 0.5\,k_\mathrm{B}T_\mathrm{K}$ [38], we would expect to observe no splitting below $B \approx 6$ T, contrary to the observed data where we see a clear splitting at $B = 2$ T (Fig. 4a main paper).

Supplementary Note 3. LINESHAPE FOR PARALLEL TUNNELING CHANNELS

The Kondo resonance found on single magnetic adatoms on noble metal surface is usually well described by a Fano function rather than a Lorentzian or Frota function, because the electrons from the tip tunnel predominantly into the conduction band rather than into the Kondo state. Analogous to the calculations of the line shape for a Lorentzian [27, 39], we can obtain the line shape in case of Kondo effect in the weak coupling regime if the tip couples only weakly to the impurity state. The local density of states due to the presence of the spin at zero field is given by

$$\rho(\epsilon) \propto -\int_{-\omega_0}^{\omega_0} \frac{f(\epsilon', T)}{\epsilon - \epsilon'} \mathrm{d}\epsilon'. \tag{S7}$$

Hence we can calculate the impurities Green's function to

$$G_\mathrm{imp}(\epsilon) = \mathrm{P}\int_{-\infty}^{\infty} \frac{\rho(\epsilon)}{\epsilon - \epsilon'} \mathrm{d}\epsilon' + \mathrm{i}\pi\rho(\epsilon), \tag{S8}$$

with $\mathrm{P}\int$ as the principal value integral.

The real part of the Green's function is essentially a step function, for $\omega_0 \to \infty$, one would obtain $\mathrm{Re}G(\omega) \propto f(\omega, T)$. From the Green's function, the change in conductance due to the impurity can be obtained [27]

$$\delta G \propto (q^2 - 1)\Im G_{\mathrm{imp}} + 2q\Re G_{\mathrm{imp}}. \tag{S9}$$

For metal adatoms on metal surfaces, typically $q \approx 0$ has been found, in which case δG becomes a logarithmic *dip*. For $q \approx 1$, the line shape becomes asymmetric, and for $q \to \infty$, the logarithmic peak is recovered (see Supplementary Figure S4). Two physical processes control the value of q: neglecting tunneling to the impurity state, it is controlled by the position of the impurity state within the conduction band [27]; if tunneling to the impurity state cannot be neglected as in the case here, the stronger direct tunneling is the closer the line shape comes to the density of states of the impurity state [39]. Our results indicate that we have a strong direct tunneling channel and only small contributions from channels which go to the conduction band.

Supplementary References

[34] J. Lambe and R. C. Jaklevic. Molecular vibration spectra by inelastic electron tunneling. *Phys. Rev.* **165**, 821-832 (1968).

[35] A. F. G. Wyatt and R. H. Wallis. Exchange scattering in Ti-doped Al/Al oxide/Ag tunnel junctions. I. Zero magnetic field. *J. Phys. C.: Solid State Phys.* **7**, 1279-1292 (1973).

[36] S. Loth, C. P. Lutz, and A. J. Heinrich. Spin-polarized spin excitation spectroscopy. *New J. Phys.* **12**, 125021 (2010).

[37] R. Zitko. Kondo resonance lineshape of magnetic adatoms on decoupling layers. *Phys. Rev. B* **84**, 195116 (2011).

[38] R. Zitko, R. Peters, and Th. Pruschke. Splitting of the Kondo resonance in anisotropic magnetic impurities on surfaces. *New J. Phys.* **11**, 053003 (2009).

[39] M. Plihal and J. W. Gadzuk. Nonequilibrium theory of scanning tunneling spectroscopy via adsorbate resonances: Nonmagnetic and Kondo impurities. *Phys. Rev. B* **63**, 085404 (2001).
